# Lipoprotein(a) Where Do We Stand? From the Physiopathology to Innovative Terapy

**DOI:** 10.3390/biomedicines9070838

**Published:** 2021-07-19

**Authors:** Gabriella Iannuzzo, Maria Tripaldella, Vania Mallardo, Mena Morgillo, Nicoletta Vitelli, Arcangelo Iannuzzi, Emilio Aliberti, Francesco Giallauria, Anna Tramontano, Raffaele Carluccio, Ilenia Calcaterra, Matteo Nicola Dario Di Minno, Marco Gentile

**Affiliations:** 1 Department of Clinical Medicine and Surgery, “Federico II” University, 80131 Naples, Italy; mariatripaldella@gmail.com (M.T.); vania.mallardo@virgilio.it (V.M.); mena.morgillo@virgilio.it (M.M.); nicoletta.vitelli@gmail.com (N.V.); ilenialorenza.calcaterra@unina.it (I.C.); dario.diminno@hotmail.it (M.N.D.D.M.); margenti@unina.it (M.G.); 2 Department of Medicine and Medical Specialties, A. Cardarelli Hospital, 80131 Naples, Italy; lelliann@alice.it; 3 North Tees University Hospital Stockton-on-Tees, Stockton TS19 8PE, UK; ealiberti@hotmail.co.uk; 4 Department of Translational Medical Sciences, “Federico II” University of Naples, Via S. Pansini 5, 80131 Naples, Italy; giallauriafrancesco@gmail.com (F.G.); annatramontano3@gmail.com (A.T.); raffaelecarluccio92@virgilio.it (R.C.)

**Keywords:** lipoprotein(a), cardiovascular risk, lipoprotein apheresis, antisense oligonucleotide APO(a)L_rx_

## Abstract

A number of epidemiologic studies have demonstrated a strong association between increasing lipoprotein a [Lp(a)] and cardiovascular disease. This correlation was demonstrated independent of other known cardiovascular (CV) risk factors. Screening for Lp(a) in the general population is not recommended, although Lp(a) levels are predominantly genetically determined so a single assessment is needed to identify patients at risk. In 2019 ESC/EAS guidelines recommend Lp(a) measurement at least once a lifetime, fo subjects at very high and high CV risk and those with a family history of premature cardiovascular disease, to reclassify patients with borderline risk. As concerning medications, statins play a key role in lipid lowering therapy, but present poor efficacy on Lp(a) levels. Actually, treatment options for elevated serum levels of Lp(a) are very limited. Apheresis is the most effective and well tolerated treatment in patients with high levels of Lp(a). However, promising new therapies, in particular antisense oligonucleotides have showed to be able to significantly reduce Lp(a) in phase II RCT. This review provides an overview of the biology and epidemiology of Lp(a), with a view to future therapies.

## 1. Introduction

Cardiovascular disease (CVD) is still the principal cause of death in the world [1,2], despite the decrease in mortality rate due to progress in its diagnosis, treatment and prevention.

Atherosclerosis is a chronic and progressive inflammatory process beginning at youth or during the second decade of life, with later clinical expression during middle or senior age [3]. It develops in different steps, through lipid storage (above all free cholesterol and cholesterol esters) in the artery wall and local inflammation leading to creation of “atherosclerotic plaque”, with partial or total vessel occlusion or thrombosis related to its rupture or erosion.

The main risk factors for atherosclerotic disease are classified as modifiable (hypercholesterolemia, diabetes mellitus, arterial hypertension, low levels of HDL cholesterol), unmodifiable (age, sex, familiarity and genetic heritage), and habits (smoking, obesity and sedentary lifestyle). The slow and progressive evolution of the atherosclerotic process underlines the importance of the early identification of patients at risk [3,4].

Recently, authors have found a strong association between high Lipoprotein(a) [Lp(a)] concentration and coronary heart disease, aortic valvular stenosis, myocardial infarction and ischemic stroke. It is considered an independent and unmodifiable risk factor [3,5].

Although clinical data underline the importance of reducing Lp(a) in CV risk reduction, only recently have clinical trials designed “ad hoc” for Lp(a) started, therefore, the current gold standard for treatment of Lp(a) hyperlipoproteinemia is still lipoprotein apheresis.

In this review, we present an update on Lp(a) metabolism, physiopathological aspects, and current and future perspectives for treatment.

## 2. Lp(a) Molecule: Structure and Metabolism

Lp(a) is a cholesterol containing lipoprotein including a cholesterol rich, LDL like particle, apolipoprotein B100 (apo B100), covalently bound to an additional apolipoprotein called apolipoprotein a (apo a) [6]; this particular and complicated feature makes it completely different from low density lipoprotein cholesterol (LDL-C) [7].

Lp(a) was first discovered by the Norwegian physician Kare Berg in 1963, during a rabbit immunization experiment with LDL lipoproteins, and initially considered as an antigenic variant of LDL-C due to the presence of an antigen called Lp(a). In the 1970s, a Swedish researcher identified a new electrophoretic lipoprotein variant associated with cardiovascular disease, initially called Lp prebeta−1 and later identified as Lp(a) [7,8].

Apo(a) is a polymorphic glycoprotein with a structural homology to plasminogen synthetized and secreted from the liver. It is covalently linked to apo-B100 through a disulphide bond between apo-B cysteine 4326 and apo(a) 4057 cysteine [5] located on the terminal side of apo-B100; consequently, apo-B48 is not involved. Lp(a) synthesis occurs in the liver, regulated by the LPA gene with dominant inheritance, with minimal or no influence of dietary and environmental factors [5]. The *LPA* gene is located at positions 26 and 27 on the long arm of chromosome 6 (6q26-27) [8]. It is described as one of the most important monogenic risk factors for cardiovascular disease [8,9]. The *LPA* gene evolves through replication and modification of plasminogen gene [7]. It is highly polymorphic with variable exonic repetition coding for a protein domain called “kringle” (K). Plasminogen presents a five triloop structure called kringles (KI, KII, KIII, KIV, KV), and a protease domain. In contrast to plasminogen, apo(a) lacks KI, KII and KIII and presents only one copy of KV, one inactive serine protease like domain, and ten subtypes of KIV (KIV1 a KIV10) due to different amino-acid replacements with a predominance of the KIV2 subtype repeated in multiple copies, which are responsible for a substantially different size heterogeneity of apo(a) isoforms inversely related to Lp(a) levels [10]. Up to 80% of patients present two different apo(a) isoforms inherited from each parent [10]; high concentrations of the smaller Lp(a) isoform, with few copies of KIV2, are strongly related to increased cardiovascular risk [9,10,11,12,13].

Apo(a) synthesis and secretion occur in the hepatocyte through different steps: transcription of the apo(a) gene, translation (identified as the crucial step in secretion rate) and posttranslational modifications needed the for folding of apo(a) [7].

Lp(a) shows apo(a) and apo(b) in a molar ratio of 1:1; patients heterozygous for two apo(a) isoforms of different size present two different particles in plasma [4,14]. Apo(a) is predominantly linked to LDL and only 2–5% of apo(a) present in the plasma is free [7]. Assembly of Lp(a) develops in two steps: firstly, a noncovalent bond between apo(a) cysteine (Cys-4057) located at KIV 3–7 and the only free cysteine of apo-B100, and after a covalent disulfide bond between the KIV9 of apo(a) and apo-B100 [7]. The site of assembly is still controversial, but the main theories describe it as on the hepatocyte surface or in the space of Disse [8]. White and Lanford, using cultures of baboon liver cells, proved that assembly occurs in extracellular space because it was inhibited by the inoculation of anti-apo(a) serum in the cultures; in contrast Frank theorized that the assembly may occur in plasma or interstitial space: he described the creation of a Lp(a) molecule similar to the native one through mixing in vitro recombinant apo(a) and LDL particles [7]. Lp(a) clearance is still one of the most important and debated targets of Lp(a) lowering therapy. Lp(a), because of its peculiar structure, can interact with different receptors. Multiple evidence have shown that five key classes of receptors are involved in the uptake of Lp(a): LDL receptors (LDLR), scavenger receptors, toll like receptors, carbohydrate receptors (lectins), and plasminogen receptors [12,13,14]. LDLR are involved in the uptake and metabolism of chylomicrons, very low density lipoprotein (VLDL), and LDL. The similarity between Lp(a) and LDL makes LDLR itself the first investigated candidate in Lp(a) clearance, with conflicting results. Previous studies conducted on human fibroblasts described Lp(a) interaction with LDLR, but many kinetic studies demonstrate that LP(a) has a longer circulating time than LDL, probably due to its small affinity with LDLR [8], related to apo(a) particle interfering in receptor binding. Statins inhibit 3-hydroxy-3-methyglutaryl coenzyme A reductase and increase LDLR gene expression with no effect in reducing Lp(a); on the contrary, a systematic review of clinical studies describes increased Lp(a) levels in patients on statin therapy [11,15].

However, recent clinical trials describe Lp(a) reduction in patients treated with PCSK9 inhibitors, probably related to LDLR becoming more effective in a setting of low LDL-C [7].

VLDL receptors (VLDLR), LDL receptor-related protein 1 (LRP1) and LDL receptor-related protein 2 (LRP2), also called “megaline”, are involved in Lp(a) uptake [11]. Competition assays show apo(a) interaction with VLDLR present on skeletal muscle, brain, heart, adipose tissue, macrophages and endothelial cells, but not in livers, so it does not seem to have an important role in Lp(a) clearance [11]. LRP1 is highly expressed in livers, playing an important role in chylomicron-remnant-metabolism through apo-E binding; it is also involved in Lp(a) uptake through its interaction with the α2 macroglobulin present in Lp(a) [11].

On the contrary, LRP2 is highly expressed in kidneys and it is probably involved in the renal clearance of Lp(a) [12]. Furthermore, all members of the LDLR family are degraded by PCSK9 protein, justifying the mild efficacy of PCSK9 inhibitors in Lp(a) reduction [11].

Toll like receptors (TLRs) and scavenger receptors are expressed on active immune cells. They promote cytokine release and inflammation through interaction with oxidized phospholipids contained in Lp(a) [OxLp(a)] [4,11]

Asialoglycoprotein receptor 1 (ASGPR1) and galectin-1, members of lectin family, interact with the desialylated parts of apo(a) [11]. Lp(a) also binds plasminogen receptors, fibrins, tissue plasminogen activators, and tissue factor pathway inhibitors. Recently, Sharm et al., in a study conducted in vitro, described plasminogen receptor Plgrkt interaction with the lysine binding site in KIV10 of Lp(a), confirming the role of the liver in Lp(a) clearance, however, its role is yet to be tested in vivo. [11,16,17]. Moreover, several studies conducted in vivo described kidney’s involvement in Lp(a) metabolism. Kidneys excrete apo(a) particles at a rate of 1–1.5 mg/dL. Patients with a low clearance rate or with end stage renal disease undergoing haemodialysis show higher Lp(a) and a reduction of urinary excretion of apo(a) [8], which begins at a glomerular filtration rate of 70 mL/min/mq.

**Figure 1:**
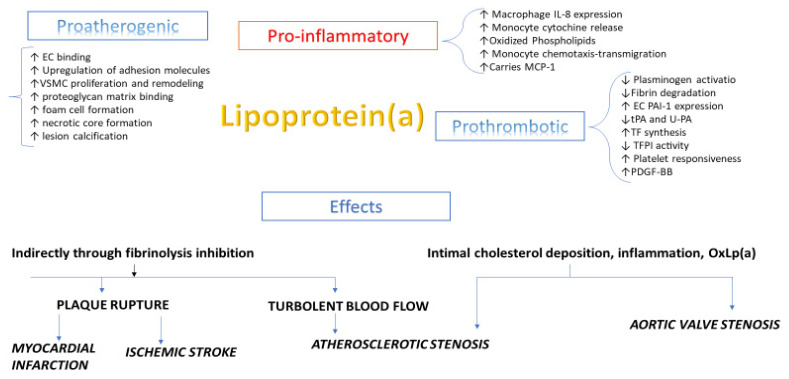
Physiological functions and pathogenicity of Lp(a).

The physiopathological role of Lp(a) is shown in Figure 1. Many investigators have reported its significant role in angiogenesis, tumour growth and wound healing [6,7]. probably due to its homology to plasminogen and through MMPS activation needed for angiogenesis; moreover, Lp(a) has shown a positive role in tissue regeneration and repair: in fact, it is present in healing tissue, particularly in the fibrous cap surface, in endothelial cells of small vessels and in extracellular space in the second stage of wound healing [7].

Authors have also described Lp(a) as an acute phase reactant with increased levels in acute illnesses such as myocardial infarction, inflammatory bowel disease, gallbladder fistula, sepsis, etc. Thus, it is mandatory to consider these clinical features in Lp(a) assay results evaluation [2,7]. Furthermore, several studies have described Lp(a) involvement in the binding and clearance of oxidized phospholipids (OxPLs), with a protective role in oxidative damage [5,7]. Lp(a) also plays a role in coagulation cascade due to its structural and functional homology with plasminogen. Coagulation is a perfect balance between procoagulant and anticoagulant factors. Vascular wall injury is a trigger for the beginning of coagulative cascade, leading to the creation of clots stabilized by fibrin cross-linking, while fibrinolysis is a controlled process leading to their suspension [7]. Plasminogen binding to fibrin promotes fibrinolysis leading to plasmin creation, which is subsequently involved in fibrin clots degradation [5].

As mentioned above, the structural homology of Lp(a) with plasminogen led to theories about its prothrombotic effect. Lp(a), through KIV5-9 and KV, binds fibrin, making up a quaternary complex that blocks new plasminogen binding and activation and its interaction with fibrin [5]. This antifibrinolytic effect is strictly related with apo(a) size, with the smaller isoform presenting a higher effect. Moreover, Lp(a) increases PAI-1 expression, resulting in the inhibition of tissue plasminogen activators (t-PA) and urinary-type plasminogen activators. Lp(a) also interacts with other prothrombotic proteins, such as α2macroglobulin (direct plasmin inhibitor) and Serpina (t-PA inhibitor), increases tissue factor (TF) synthesis and reduces TFPI (tissue factor pathway inhibitor) inhibition [18]. Concerning the role of Lp(a) in atherosclerosis, many studies conducted in vivo using radiolabelled Lp(a) have described that it may penetrate the vascular wall at a similar rate to LDL-C, with different features: LDL can enter both healthy and atherosclerotic vessels when its plasma levels reach a certain threshold, as low as 60 mg/dL, on the other hand, Lp(a) can enter only the atherosclerotic wall, exhibiting pro-inflammatory attributes, highlighting a late involvement in the atherosclerotic process, probably due to its longer circulation time [19] related to apo(a) recycling or to the selective binding of apo(a) to the matrix intima or to its interaction with SR-B1, which leads to the formation of focal deposits of Lp(a). After entering the vascular wall, ROS and lipoxygenase oxidize Lp(a), turning it into OxLp(a), which can increase both local flogosis [20,21,22,23] and endothelial permeability through Src kinase pathway, resulting also in a tight junction rupture [24]. Moreover, OxLp(a) because of its different catabolism, also increases the development of foam cells. OxLp(a) also interacts with VEGFR2 (vascular endothelial growth factor receptor 2) and increased Rho/Rho kinase activation, resulting in the phosphorylation of a myosin light chain by MLCK (Ca^2+^/calmomodulin-activated MLC kinase), which stimulates actomyosin contractility, endothelial cells retraction, and the development of intercellular fissures [25]. Lp(a) and its pathogenic fragment apo(a) may also independently induce the chemo-attraction of monocyte cells through a cGMP dependent mechanism, in addition, apo(a) interaction with β2 integrin Mac-1, which leads to monocyte transendothelial adhesion and migration, and phenotypic changes resulting in atheroma development. Advanced atherosclerotic processes lead to vascular remodelling, with vascular smooth muscles cells switching and endothelial barrier dysfunction. The aim of vascular smooth muscle cells (VSMC) remodelling in atherogenesis is to protect foam cell and create a “stable plaque” with a thick fibrous cap. Lp(a) also acts in vascular smooth muscle cells’ migration through TGF- β inhibition [7].

## 3. The Effect of “Nongenetic Factors” on Lp(a)

Lp(a) plasma levels are mainly regulated by the LPA gene, but authors also describe some other “nongenetic factors” that may influence its bloodstream concentration. As concerning environmental factors, age, sex, race and habits have been evaluated. Several studies show increased Lp(a) levels since birth, with a progressive elevation during the first eight months of life; authors also describe a positive relationship between Lp(a) and age, regardless of sex, in White and Japanese people, not confirmed in Black people [5,7]. Lp(a) also presents differences in plasma levels and the distribution of apo(a) isoforms related to LPA gene polymorphisms, with the lowest concentration in Caucasian patients and the highest in Africans [26]. As described above, acute inflammatory illnesses, such as sepsis inflammatory bowel disease, gallbladder fistula, acute myocardial infarction, present increased Lp(a) concentration with a positive relationship with IL-6, PCR and α 1 antitripsin [3,27] and return back to a normal level when acute phases withdraw. Lp(a) increases one to two fold in pregnancy with normal level after delivery [8], while women treated with hormone replacement therapy postmenopause show decreased Lp(a) concentration.

Chronic liver disease, hypothyroidism, acromegaly and diabetes are related to increased levels with a return back to habitual concentration after the improvement of clinical conditions [28,29], cholestatic disease presents decreased levels instead, probably due to a reduction in Lp(a) synthesis. On the contrary, several studies demonstrate the strong influence of ethanol and tobacco in reducing Lp(a) concentration in a dose dependent manner, regardless of different apo(a) isoforms, by up to 60% and 20%, respectively [5,7]. Obese patients, too, show decreased Lp(a) levels, probably related to insulin resistance.

It is necessary to mention the strong relationship between Lp(a) concentration in the bloodstream and glomerular filtration rate (GFR). People with chronic kidney disease show a reduction in Lp(a) clearance and, on the other hand, increased Lp(a) leads to the worsening of kidney function. A prospective study carried out on 852 patients with type 2 diabetes underlines the role of Lp(a) as an independent prognostic factor in the development of chronic kidney disease in patients with type 2 diabetes [30]. Authors also describe increased Lp(a) levels in patients with nephrotic syndrome, related not only to low GFR but also to enhanced protein synthesis due to hypoalbuminemia [29,31]. The Penn Diabetes Heart Study, conducted on 1852 patients with type 2 diabetes without clinical evidence of cardiovascular disease or kidney impaired function, described the early elevation of Lp(a) levels in patients with chronic kidney disease, even before GFR goes under 70/mL/min/mq. Moreover, Kronenberg et al. demonstrated a worsening of kidney function, despite aetiology, through evaluation of the relationship between different apo(a) isoforms, kidney function and Lp(a) levels [32]. Several studies also described the influence of haemodialysis on Lp(a) concentration in patients with end stage renal disease (ESRD): they present increased Lp(a) levels, related to a large apo(a) isoform, compared to healthy subjects, and 5 to 10 times higher than subjects with kidney disease at early stages; on the contrary, Milionis described Lp(a) elevation in patients treated with peritoneal dialysis regardless of apo(a) isoform size [33]. Authors also describe a return back to normal Lp(a) levels after kidney transplants, probably due to improved clearance [32].

## 4. Lp(a) Measurement

The peculiar Lp(a) features, such as high heterogeneity, the covalent bound between apo(a) and apoB and its homology with plasminogen, have long been a major challenge in the development of suitable and reliable immunoassay for the measurement of Lp(a). Various immunochemical methods, such as ELISA, immunoturbidimetry, nephelometry and dissociation enhanced lanthanide immunoassay were employed in Lp(a) evaluation in serum or plasma using antibodies versus apo(a). Immunoassays are based on the measurement of signals due to antigen–antibody interaction. There are two categories of immunoassays involved in Lp(a) measurement. The “isoform dependent” method evaluates the entire protein mass, reported in mg/dL, including lipids, proteins and carbohydrates, and it is strongly related to the number of KIV2 copies with a range between 200 and 800 kilodaltons [24]; extreme apo(a) isoform size variability is associated with the overestimation of Lp(a) levels in patients with large apo(a) isoforms, and the underestimation of Lp(a) concentration in those with small apo(a) isoforms [8,34], leading to incorrect cardiovascular risk assessment.

The other category, “isoform-independent”, reports Lp(a) in nmol/L; it uses antibodies binding KIV9, the unique nonrepeating kringle IV subtype and it is considered the gold standard by the International Federation of Clinical Chemistry and Laboratory Medicine (IFCC) and approved by the World Health Organization (WHO) as the preferred measuring method in Lp(a) assessment, because it is not influenced by different apo(a) sizes [7].

Concerning apo(a) isoform size, three methods are available to evaluate KIV repeats on a DNA level. Pulsed field gel electrophoresis (PFGE)/southern blotting of genomic DNA [14], through a particular DNA preparation, permits the evaluation of KIV2 number copies in separated alleles; FISH (fiber-fluorescence in situ hybridization) also allows this by fluorescence microscopy. In contrast, qPCR (quantitative polymerase chain reaction) evaluates the sum of KIV2 copies present in investigated genomes, leading to the underestimation of CV risk. Moreover, it is not recommended to use of mean conversion factor of 2.4, used in many previous studies to convert mass based concentrations, expressed in mg/dL or mg/L, to molar concentration in nmol/L, because unlike the other conversion factors used in the evaluation of analytes with a definite molecular mass, it does not consider the great heterogeneity of apo(a), leading to reduction in Lp(a) measurement test accuracy. Lp(a) also shows asymmetric distribution in populations and among different races, for this reason it is needed to establish different cut offs for different populations [35]. Recently EAS (the European Atherosclerotic society) and EFLM (the European Federation di Clinical Chemistry and Laboratory Medicine) guidelines recommend Lp(a) evaluation with lipid lowering therapy in patients with low or absent results, or in patients with no therapeutic target achievement. Therefore, the authors recommend to correct LDL-C values according to Lp(a) with one of this formula [5]:

LDL (C) adjusted for Lp(a) (mg/dL) = LDL − C(mg/dL) − [Lp(a) mg/dL × 0.30] LDL(C) adjusted for Lp(a)(mmol/L) = LDL − C(mmol/L) − [Lp(a)mmol/L × 0.0078]

In 2018, the American Heart Association/American College of Cardiology (AHA/ACC), and the European Society of Cardiology (ESC) guidelines of 2016, highlighted a threshold value of 50 mg/ dl associated with increased cardiovascular risk; however, a gradual increase in risk has already been found from values over 30 mg/dL [6].

## 5. Lp(a) Screening

In susceptible subjects, Lp(a) increases quickly after birth, reaching high and constant levels in few months [36]. Its range in adults varies from <2 to 2.500 mg/dL [37], regardless of sex, although several recent studies demonstrate a slight prevalence in women, particularly in pregnancy. Moreover, studies on twins and families underline that heritability of the quantitative Lp(a) trait, related to the LPA locus, is very high, 70 to > 90%, in all populations evaluated (Europe, Asia and Africa) [9]. Lp(a) also presents significantly different distribution in various populations, with the lowest levels in Caucasian and the highest in Africans, but there is not an established race specific clinical cut-off point. According to a meta-analysis on 56,000 subjects from 31 studies, patients with higher Lp(a) levels present a higher risk of ischemic stroke. The Copenhagen City Heart Study, published in 2008, demonstrates a relationship between elevated Lp(a) levels and an increased risk of acute myocardial infarction, although a threshold value is not precisely indicated. The study describes a 1.6-fold increased risk in individuals with Lp(a) levels between 30 and 76 mg/dL (the 67th and 90th percentiles) compared to those with Lp(a) concentration below 5 mg/dL, while patients with Lp(a) between 77 and 117 mg/dL (90–95th percentile) or Lp(a) levels above 117 mg/dL (>95th percentile) presents 1.90 and 2.60-fold increased risk, respectively, for myocardial infarction [14]. The PROCARDIS study [38,39] describes two variants of the *LPA* gene (rs104558272 and rs3798220) more closely associated with increased Lp(a) levels, which playing a causative role in the development of coronary heart disease and myocardial infarction; recently, rs10455872 was also associated with an increased risk of aortic valve stenosis [38,39].

Screening in the general population is not recommended, although Lp(a) levels are predominantly genetically determined, so a single assessment is needed to identify patients at risk [40]. In 2019, ESC/EAS guidelines recommend Lp(a) measurement at least once in a lifetime in subjects at very high and high CV risk and in those with a family history of premature cardiovascular disease, to reclassify patients with borderline risks [6]. Moreover, the National Lipid Association (NLA) and AHA/ACC (American Heart Association/American College of Cardiology) guidelines recommend Lp(a) evaluation in patients with a history of premature cardiovascular disease in first degree relatives (<55 years in men and <65 years in women) or with a personal history of premature cardiovascular disease or with recurrent cardiovascular events despite optimal lipid lowering therapy, and in patients with familial hypercholesterolemia [6].

## 6. Treatments for High Lp(a)

### 6.1. Traditional Lipid Lowering Therapies

A healthy diet and physical exercise are recommended to reduce CV risk for their favourable effect on lipid profile, but they have no effects on Lp(a) levels. In contrast, a recent study reported that a plant based diet reduces inflammatory biomarkers and atherogenic lipoproteins [41], also with a certain impact on Lp(a) concentration. Anyway, correction of lifestyles factors should also be carefully considered in the treatment of Lp(a) CVD preventive care for their effect LDL-C and HDL-C. As concerning medications (Table 1), statins play a key role in lipid lowering therapy in both primary and secondary prevention, but present poor efficacy on Lp(a) levels. Data from JUPITER (Justification for the Use of Statins in Prevention: an Intervention Trial Evaluating Rosuvastatin) also described a positive shift in Lp(a) plasma levels by 10–20% in patients on therapy with rosuvastatin, probably related to cholesterol contained in Lp(a) and not in LDL molecules [15,42]. Regardless, statins are strongly recommended for Lp(a) hyperlipoproteinemia to maximally reduce CV risk related to LDL-C [43]. Moreover, ezetimibe as a monotherapy or in combination with statins and lomitapide have no effect on Lp(a) reduction [44]. Fibrates still present doubtful results [45]. They induce PPAR α with the consequent activation of farnesoid X receptors that inhibits apo(a) transcription; moreover, the fatty acids released from adipose tissue enhances their lowering effect on Lp(a) levels with an unclear mechanism. Niacin is considered a “broad spectrum” lipid lowering drug; it reduces the mobilization of free fatty acids from adipose tissue to the liver and stimulates degradation of all apo-B-containing lipoproteins, from chylomicrons to Lp(a); it also reduces triglycerides through the inhibition of diacilglycerolacyltransferase-2 [46,47,48]. A systematic meta-analysis of head-to-head randomized controlled trials also shows that the effect of fibrates on Lp(a) is more effective in patients with elevated levels at baseline [45].

A meta-analysis on 14 randomized placebo controlled clinical trials showed LDL-C and Lp(a) reduction by 45% and 20–30%, respectively, in subjects in therapy with niacin with no CV risk reduction [7]. EAS guidelines recommend niacin at a dose of 1–3 g/day in high risk patients, despite LDL-C target achievement [7]. Anacetrapib is a CEPT (cholesteryl ester transfer protein) inhibitor that increases HDL-C and reduces LDL-C through the inhibition of the transfer of cholesterol esters [49]. A randomized clinical trial aimed to evaluate the safety and efficacy of anacetrapib on 1623 patients with cardiovascular disease shows Lp(a) reduction by 38.8% compared to baseline values, however, despite its excellent results, research activities have stopped [50].

Mipomersen is a second generation antisense oligonucleotide (ASO) inhibiting apo-B synthesis with no effect on apo(a), approved by the FDA in addition to statin therapy for the treatment of homozygous familial hypercholesterolemia [51]. It decreases Lp(a) levels by 25–40% with important side effects (site of injection reactions, hepatic steatosis, hypertransaminasemia) leading to limited therapeutic use [52]. PCSK9 inhibitors, evolocumab and alirocumab, are fully monoclonal antibodies interfering in LDL receptor “recycling”: they interact with PCSK9 proteins and reduce LDLR clearance leading, to a decrease in LDL-C of 60–70% [53]. Trials also demonstrated a constant reduction in Lp(a) levels, by 30%, with a not yet well defined mechanism [54] probably related to the effect of LDLR on Lp(a) concentration in a setting of low LDL-C. A postanalysis of the FOURIER trial shows Lp(a) reduction by 26.9% in patients on therapy with evolocumab, with more effectiveness in subjects with higher levels at baseline. Instead, the ODYSSEY OUTCOMES trial demonstrates both the reduction of Lp(a) concentration and cardiovascular events in patient on therapy with alirocumab, but it is not clear if the decrease in cardiovascular events is related to reduced Lp(a) or to low LDL-C levels [55]. Inclisiran is a long-acting siRNA that reduces PCSK9 synthesis. Results from a single blind placebo controlled phase I trial show LDL-C and Lp(a) reduction by 50% and 48.1%, respectively, with a dose of 300 mg, instead in a second phase double blind, placebo controlled trial with ascending doses, subjects treated with 200 mg present a persistent decrease in LDL-C and Lp(a) after 90 days, by 52.6% and 25.6%, respectively [56,57].

### 6.2. Apheresis

Apheresis is the most effective and well tolerated treatment in patients with Lp(a) hyperlipoproteinemia. “Apheresis” comes from the Greek “ἀφαίρεσις”, which literally means “to eliminate” or “to remove”; it plays an important role in treatment of different diseases, particularly in those conditions when pharmacological therapy is not effective, when it is needed to obtain therapeutic effects as soon as possible, or when drugs lead to serious adverse events.

Lipoprotein apheresis (LA) was first introduced as a therapeutic option in treatment of homozygous familial hypercholesterolemia in the mid-1970s.

It is based on three physical separation processes of whole blood: differential centrifugation, membrane filtration and substances and cells absorption, previously separated from whole blood or plasma.

Plasmapheresis was the first effective extracorporeal method using a nonspecific plasma separation with a subsequent replacement with fresh plasma and/or albumin. Its lack of selectivity leads to the elimination of all plasma proteins that need to be reinfused. Currently heparin mediated, extracorporeal, low density lipoprotein (LDL) fibrinogen precipitation (H.E.L.P.) and cascade filtration are the most favourite methods because they use the native venous system instead of invasive vascular access with less “stress” on different blood components, less complications related to access itself, and milder side effects, such as the nausea and hypotension described by 3.4% of treated patients [58].

Cascade filtration or “double filtration plasmapheresis” is a semiselective venous-vein system using needles between 17–19 G; it involves, firstly, plasma separation through a plasma filter, subsequent filtration through a capillary filter with a variable diameter of 0.01–0.02 microns related to different sizes of molecule to remove, and, finally, filtrated plasma is rejointed to other plasma components and returned to the patient. Filters with different porosities increase plasma treatment selectivity, saving other plasma components (coagulation factors and albumin above all). It is not recommended in patients on therapy with ace inhibitors because of the high risk of anaphylactoid reactions and severe hypotension related to the increased release of bradichinin. H.E.L.P was first introduced in 1983. Plasma is first separated by a polypropylene plasma filter and later treated with an acetate–heparin buffer solution at a pH of 4.85, leading to lipoprotein and fibrinogen precipitation. Pellet is removed from plasma by polycarbonate or polysulfone filters; later, another dialysis filter and a bicarbonate buffer solution remove heparin excess and restore physiological pH before pooling it with other blood components and patient reinfusion.

The H.E.L.P. technique treats about 3 plasma litres with a blood flow of 50–100 mL/min (plasma flow of 20–35 mL/min) in 3 h [59]. H.E.L.P. and cascade filtration both reduce LDL-C by 60% (4% more achieved with H.EL.P.) and Lp(a) by 55–60% and 52%, respectively, in a single session, while HDL-C is reduced by 5% and 20%, respectively [59]. Moreover, H.E.L.P. is more effective in the removal of inflammation molecules; in a single session, it can reduce oxLDL by 48%, PCR and adhesion tissue molecules by 57–59% and 20, respectively, particularly E-selectin [60,61,62], tissue factor and CD 40 [59,61]. Moreover, Morawietz et al. also described a decreased expression of ox-LDLR and VCAM-1 and increased expression of e-NOS in patients on lipoprotein apheresis [63]. In 2009, an Italian multicentric study demonstrated that long term apheresis stopped the evolution of cardiovascular disease in high risk patients with Lp(a) hyperlipoproteinemia. A longitudinal cohort multicentric study on 120 subjects with coronary heart disease and Lp(a) hyperlipoproteinemia, treated with apheresis and maximum lipid lowering therapy, showed Lp(a) reduction by 73.3% with a subsequent decrease in annual cardiovascular CV events by 86.4% [64]. In 2010, Stefanutti et al. [65], in a study which enrolled 21 patients with documented coronary artery disease (CAD), demonstrated Lp(a) reduction by 57.8 ± 9.5% in patients on apheresis and Lp(a) increase by 14.7 ± 36.5% in a control group.

Currently, the FDA approves lipoprotein apheresis for homozygous familial hypercholesterolemia (HoFH), with LDLC > 500 mg/dL, heterozygotes familial hypercholesterolemia (HeFh) with LDL > 300 mg/dL or LDL > 200 mg in primary or secondary prevention, respectively. Moreover, in 2008, The Federal Joint Committee (G-BA) recommended lipoprotein apheresis A for HoFH, in HeFh patients in secondary prevention with LDL > 120 mg/dL despite maximum lipid lowering pharmacological therapy, and in subjects with increased Lp(a) levels > 60 mg/dL (120 nmol/L) with documented cardiovascular disease progression [66]; in Japan it is approved for patients with previous CV events with LDL-C > 250 mg/dL [49]. The American Society for Apheresis recommends it for Lp(a) hyperlipoproteinemia, although it is used infrequently because of its expensive costs. In Italy, Lp(a) hyperlipoproteinemia is recognized as indication for apheresis in patients with premature cardiovascular disease or in secondary prevention [65].

### 6.3. Future Therapies

None of the mentioned pharmacological therapies is able to reduce Lp(a) levels significantly. Aphaeretic treatment is not always available, and it should be performed in dedicated centres. On the other hand, the recent introduction in clinical practice of antisense oligonucleotide (ASO) and data from preclinical proof of concept studies led to the development of ASOs targeting hepatic the LPA messenger RNA (mRNA) to exclusively reduce plasma levels of lipoprotein(a), PMID: 31893580.

Data from phase 1 and 2 studies of a non–hepatocyte-targeted, second generation ASO showed efficacy in Lp(a) lowering both in healthy participants and in patients with CV disease and high levels of Lp(a). Advances in ASO development, in particular directing ASOs to hepatocytes by conjugation with a triantennary N-acetylgalactosamine (GalNAc3) [67], improved affinity for hepatocyte specific asialoglycoprotein receptors (ASGPR), ameliorating drug distribution in hepatocytes and drug selectivity [68].

ISIS 681257 also named IONIS APO(a)Lrx as the new GalNAc3 ASO targeting apo(a) synthesis in hepatocyte. ISIS 681257 acts like a prodrug converted in active drug once internalized. After subcutaneous administration, it binds plasma proteins and enters the liver where ASO is separated from GalNAc sugars; after intracellular collection, it selectively binds apo(a) mRNA in the nucleus, leading to antisense RNA complex degradation by RNase H1. After single subcutaneous administration, it is quickly cleared from plasma within 4 h through liver uptake, it is cleaved and turned into unconjugated ISIS 681257 which is cleared from tissue with a half-life of 6–9 days. Results from phase 1 clinical trials for the safety and efficacy of IONIS APO(a)-Lrx in healthy subjects showed that multiple doses (19 mg–20 mg–40 mg) can reduce Lp(a) plasma concentration by 66%, 80% and 92%, respectively, in three weeks. Results of phase 2b RCT on 286 patients with established CVD (CAD, previous IMA, PAD, ischemic stroke) disease and increased Lp(a) levels (>60 mg/dL) evidenced a percentage change from baseline after 6 months, by 35% with 20 mg/4 W, by 56% with a dose of 40 mg/4 W, and 72% in patient treated with 60 mg/4 W, instead, a reduction by 58% and 80% was found with 20 mg/2 W and 20 mg/4 W, respectively. No serious adverse event was reported in all groups, and the most frequently described side effect was reaction at injection site. Furthermore, a phase 3, randomized, double-blind, placebo controlled, multicentre trial assessing the impact of Lp(a) lowering with TQJ230 on major cardiovascular events in patients with established CV disease study is currently on active recruitment [69].

## 7. Conclusions

Lp(a) is considered as an independent and unmodifiable risk factor for coronary heart disease, aortic valve stenosis, myocardial infarction and ischemic stroke, and recognized as an additional risk factor in patients with LDL-C achieved target levels. Lp(a) concentration is strongly genetically determined and its homology to plasminogen enhances its atherogenicity. Moreover, in contrast to other lipoproteins characterized by constant masses, Lp(a) also presents different isoforms of various sizes inversely related to its plasma concentration [70].

For this peculiar feature, Lp(a) measurement presents many challenges and the development of isoform independent assays targeting KIV9 improves its evaluation in a significant way. Although clinical data underline the importance of reducing Lp(a) in cardiovascular risk reduction, there is not a selective drug approved for Lp(a) hyperlipoproteinemia. Apheresis, especially the H.EL.P. technique, decreases both Lp(a) and LDL-C through the elimination of proteins containing apo-B. It is recognized as a gold standard therapy in Lp(a) hyperlipoproteinemia. Anyway, lipoprotein apheresis does not reduce Lp(a) permanently, and needs to be repeated every two weeks. Recently, data from clinical trials evaluating the efficacy and safety of IONIS-APO(a)-Lrx, targeting apo(a) mRNA, shows encouraging results and represents a hopeful focused Lp(a) lowering therapy which could finally replace the role of lipoprotein apheresis.

In conclusion, Lp(a) is a cholesterol molecule involved in cardiovascular disease, alone or associated to other cardiovascular risk factors. Several epidemiological studies have highlighted Lp(a) as one of the major risk factors for CVD, even in patients on target therapy. Actually, there are only a few Lp(a) lowering therapies and clinical trials evaluating the role of ASOs targeting Lp(a) that have shown promise for the future.

## Figures and Tables

**Table 1:** Summary of approved or investigational therapeutic drug to lower Lp(a) and LDL-C.

Approved and Investigational Drugs to Lower Lp(a) and LDL-C					
Mechanism	Agent	Lp(a) Δ%	LDL-C Δ%	STATUS	Specific for Lp(a)
Reduce production of new Lp(a)/LDL-C	Statins	↑ 0–20	↓ 19–60	Approved	no
	Niacin	↓ 30–40	↓ >45	Approved	no
	Fibrates	-	↓ 10–30	Approved	no
	EPA/DHA	-	-	Approved	no
	Probucol	-	↓ 11–33	Approved	no
	Mipomersen	↓ 20–33	↓ 21–40	Approved	no
	IONIS APO(a)Lrx	↓ 39–92	-	Investigational	yes
	Lomitapide	↓ 19–51	↓ 15–15	Approved	no
	CEPT inhibitors	↓ 14–26	-	Stopped	no
	Aspirin	↓ 10–80	-	Approved	no
Increase LDLR expression for Lp(a)/LDL uptake	Evolocumab	↓ 30	↓ 39–75	Approved	no
	Alirocumab	↓ 30	↓ 29–73	Approved	no
	Inclisiran	↓ 26	↓ >60	Investigational	no

↑ increasing value; ↓ decreasing value.

## Data Availability

Not applicable.

## References

[1] Barquera S., Pedroza-Tobías A., Medina C., Hernández-Barrera L., Bibbins-Domingo K., Lozano R., Moran A.E. (2015). Global Overview of the Epidemiology of Atherosclerotic Cardiovascular Disease. Arch. Med. Res..

[2] Giordano A., Peruzzi M., Marullo A.G.M., Frati G., Sciarretta S., Napolitano G., Biondi-Zoccai G. (2017). What We Learned with Recent Network Meta-analyses on Atherosclerosis Prevention and Treatment. Curr. Atheroscler. Rep..

[3] Gentile M., Iannuzzo G., Mattiello A., Marotta G., Iannuzzi A., Panico S., Rubba P. (2016). Association between Lp(a) and atherosclerosis in menopausal women without metabolic syndrome. Biomarkers Med..

[4] Gentile M., Simeon V., Iannuzzo G., Mattiello A., di Taranto M.D., Panico S., Rubba P. (2020). Lipoprotein(a) is an independent predictor of cardiovascular events in Mediterranean women (Progetto Atena). Eur. J. Prev. Cardiol..

[5] Shah N.P., Pajidipati N.J., McGarrah R.W., Navar A.M., Vemulapalli S., Blazing M.A., Shah S.H., Hernandez A.F., Patel M.R. (2020). Lipoprotein(a): An Update on a Marker of Residual Risk and Associated Clinical Manifestations. Am. J. Cardiol..

[6] Mach F., Baigent C., Catapano A.L., Koskinas K.C., Casula M., Badimon L., Chapman M.J., De Backer G.G., Delgado V., Ference B.A. (2020). 2019 ESC/EAS Guidelines for the management of dyslipidaemias: Lipid *modification to reduce cardiovascular risk*: The Task Force for the management of dyslipidaemias of the European Society of Cardiology (ESC) and European Atherosclerosis Society (EAS). Eur. Heart J..

[7] Jawi M.M., Frohlich J., Chan S.Y. (2020). Lipoprotein(a) the Insurgent: A New Insight into the Structure, Function, Metabolism, Pathogenicity, and Medications Affecting Lipoprotein(a) Molecule. J. Lipids.

[8] Tsimikas S. (2017). A test in context: Lipoprotein(a): Diagnosis, prognosis, controversies, and emerging therapies. J. Am. Coll. Cardiol..

[9] Schmidt K., Noureen A., Kronenberg F., Utermann G. (2016). Structure, function, and genetics of Lipoprotein(a). J. Lipid Res..

[10] Enkhmaa B., Anuurad E., Berglund L. (2016). Lipoprotein(a): Impact by ethnicity and environmental and medical conditions. J. Lipid Res..

[11] Sally P., McCormick A., Schneider W. (2019). Lipoprotein(a) catabolism: A case of multiple receptors. Pathology.

[12] Berman A.N., Blankstein R. (2019). Current and future role of lipoprotein(a) in preventive cardiology. Curr. Opin. Cardiol..

[13] Tada H., Takamura M., Kawashiri M.-A. (2019). Lipoprotein(a) as an Old and New Causal Risk Factor of Atherosclerotic Cardiovascular Disease. J. Atheroscler. Thromb..

[14] Kronenberg F. (2019). Prediction of cardiovascular risk by Lp(a) concentrations or genetic variants within the LPA gene region. Clin. Res. Cardiol. Suppl..

[15] Yeang C., Hung M.-Y., Byun Y.-S., Clopton P., Yang X., Witztum J.L., Tsimikas S. (2016). Effect of therapeutic interventions on oxidized phospholipids on apolipoprotein B100 and lipoprotein(a). J. Clin. Lipidol..

[16] Miles L.A., Baik N., Lighvani S., Khaldoyanidi S., Varki N.M., Bai H., Mueller B., Parmer R.J. (2017). Deficiency of plasminogen receptor, Plg-RKT, causes defects in plasminogen binding and inflammatory macrophage recruitmentin vivo. J. Thromb. Haemost..

[17] Sharma M., Redpath G., Williams M.J., McCormick S.P. (2017). Recycling of Apolipoprotein(a) After PlgRKT-Mediated Endocytosis of Lipoprotein(a). Circ. Res..

[18] Von Zychlinski A., Kleffmann T., Williams M.J., McCormick S.P. (2011). Proteomics of Lipoprotein(a) identifies a protein complement associated with response to wounding. J. Proteom..

[19] Nordestgaard B.G., Langsted A. (2016). Lipoprotein(a) as a cause of cardiovascular disease: Insights from epidemiology, genetics, and biology. J. Lipid Res..

[20] Orsó E., Schmitz G. (2017). Lipoprotein(a) and its role in inflammation, atherosclerosis and malignancies. Clin. Res. Cardiol. Suppl..

[21] Pirro M., Bianconi V., Paciullo F., Mannarino M.R., Bagaglia F., Sahebkar A. (2017). Lipoprotein(a) and inflammation: A dangerous duet leading to endothelial loss of integrity. Pharmacol. Res..

[22] Van Der Valk F.M., Bekkering S., Kroon J., Yeang C., Bossche J.V.D., Van Buul J.D., Ravandi A., Nederveen A.J., Verberne H.J., Scipione C. (2016). Oxidized Phospholipids on Lipoprotein(a) Elicit Arterial Wall Inflammation and an Inflammatory Monocyte Response in Humans. Circulation.

[23] Riches K., Porter K.E. (2012). Lipoprotein(a): Cellular Effects and Molecular Mechanisms. Cholesterol.

[24] García-Gómez C., Bianchi M., de la Fuente D., Badimon L., Padró T., Corbella E., Pintó X. (2014). Inflammation, lipid metabolism and cardiovascular risk in rheumatoid arthritis: A qualitative relationship?. World J. Orthop..

[25] Birukova A.A., Lee S., Starosta V., Wu T., Ho T., Kim J., Berliner J.A., Birukov K.G. (2012). A Role for VEGFR2 Activation in Endothelial Responses Caused by Barrier Disruptive OxPAPC Concentrations. PLoS ONE.

[26] Marcovina S.M., Albers J.J. (2016). Lipoprotein(a) measurements for clinical application. J. Lipid Res..

[27] Maranhão R.C., Carvalho P.O., Strunz C., Pileggi F. (2014). Lipoprotein(a): Structure, Pathophysiology and Clinical Implications. Arq. Bras. Cardiol..

[28] Yin D., Shao P., Liu Y. (2016). Elevated Lipoprotein(a) levels predict deep vein thrombosis in acute ischemic stroke patients. NeuroReport.

[29] Kronenberg F. (2013). Causes and consequences of lipoprotein(a) abnormalities in kidney disease. Clin. Exp. Nephrol..

[30] Yun J.-S., Ahn Y.-B., Song K.-H., Yoo K.-D., Park Y.-M., Kim H.-W., Ko S.-H. (2015). Lipoprotein(a) predicts a new onset of chronic kidney disease in people with Type 2 diabetes mellitus. Diabet. Med..

[31] Hopewell J.C., Haynes R., Baigent C. (2018). The role of Lipoprotein(a) in chronic kidney disease. J. Lipid Res..

[32] Kon V., Yang H., Fazio S. (2015). Residual Cardiovascular Risk in Chronic Kidney Disease: Role of High-density Lipoprotein. Arch. Med. Res..

[33] Gambhir J.K., Kalra O.P., Khaira A., Kaur H. (2013). Association between high molecular weight apolipoprotein isoforms and lipoprotein levels in advanced chronic kidney disease and the effect of hemodialysis. Indian J. Nephrol..

[34] Pavanello C., Pirazzi C., Bjorkman K., Sandstedt J., Tarlarini C., Mosca L., Romeo S., Calabresi L., Mancina R.M. (2019). Individuals with familial hypercholesterolemia and cardiovascular events have higher circulating Lp(a) levels. J. Clin. Lipidol..

[35] Kamstrup P.R., Tybjærg-Hansen A., Nordestgaard B.G. (2013). Extreme Lipoprotein(a) levels and improved cardiovascular risk prediction. J. Am. Coll. Cardiol..

[36] Tsimikas S., Hall J.L. (2012). Lipoprotein(a) as a potential causal genetic risk factor of cardiovascular disease: A rationale for in-creased efforts to understand its pathophysiology and develop targeted therapies. J. Am. Coll. Cardiol..

[37] Bucci M., Tana C., Giamberardino M., Cipollone F., Bucci M., Tana C., Giamberardino M., Cipollone F. (2016). Lp(a) and cardiovascular risk: Investigating the hidden side of the moon. Nutr. Metab. Cardiovasc. Dis..

[38] Thanassoulis G., Campbell C.Y., Owens D., Smith J.G., Smith A.V., Peloso G.M., Kerr K., Pechlivanis S., Budoff M.J., Harris T.B. (2013). Genetic Associations with Valvular Calcification and Aortic Stenosis. N. Engl. J. Med..

[39] Kamstrup P.R., Tybjærg-Hansen A., Nordestgaard B.G. (2014). Elevated Lipoprotein(a) and Risk of Aortic Valve Stenosis in the General Population. J. Am. Coll. Cardiol..

[40] Nordestgaard B.G. (2017). A test in context: Lipid profile, fasting versus nonfasting. J. Am. Coll. Cardiol..

[41] Najjar R.S., Moore C.E., Montgomery B.D. (2018). Consumption of a defined, plant-based diet reduces lipoprotein(a), inflammation, and other atherogenic lipoproteins and particles within 4 weeks. Clin. Cardiol..

[42] Albers J.J., Slee A., O’Brien K.D., Robinson J.G., Kashyap M.L., Kwiterovich P.O., Xu P., Marcovina S.M. (2013). Relationship of apolipoproteins A-1 and B, and Lipoprotein(a) to cardiovascular outcomes: The AIM-HIGH trial (Atherothrombosis Intervention in Metabolic Syndrome with Low HDL/High Triglyceride and Impact on Global Health Outcomes). J. Am. Coll. Cardiol..

[43] Sahebkar A., Simental-Mendía L.E., Pirro M., Banach M., Watts G.F., Sirotri C., Al-Rasadi K., Atkin S.L. (2018). Impact of ezetimibe on plasma lipoprotein(a) concentrations as monotherapy or in combination with statins: A systematic review and meta-analysis of randomized controlled trials. Sci. Rep..

[44] Fras Z. (2019). Increased cardiovascular risk associated to hyperlipoproteinemia (a) and the challenges of current and future therapeutic possibilities. Anatol. J. Cardiol..

[45] Sahebkar A., Simental-Mendía L.E., Watts G., Serban M.-C., Banach M., Lipid and Blood Pressure Meta-analysis Collaboration (LBPMC) Group (2017). Comparison of the effects of fibrates versus statins on plasma lipoprotein(a) concentrations: A systematic review and meta-analysis of head-to-head randomized controlled trials. BMC Med..

[46] Jacobson T.A. (2013). Lipoprotein(a), Cardiovascular Disease, and Contemporary Management. Mayo Clin. Proc..

[47] Gencer B., Kronenberg F., Stroes E.S., Mach F. (2017). Lipoprotein(a): The revenant. Eur. Heart J..

[48] Schwartz J., Padmanabhan A., Aqui N., Balogun R.A., Connelly-Smith L., Delaney M., Dunbar N.M., Witt V., Wu Y., Shaz B.H. (2016). Guidelines on the Use of Therapeutic Apheresis in Clinical Practice-Evidence-Based Approach from the Writing Committee of the American Society for Apheresis: The Seventh Special Issue. J. Clin. Apher..

[49] Vogt A. (2017). Lipoprotein(a)-apheresis in the light of new drug developments. Atheroscler. Suppl..

[50] Cannon C.P., Shah S., Dansky H.M., Davidson M., Brinton E.A., Gotto A.M., Stepanavage M., Liu S.X., Gibbons P., Ashraf T.B. (2010). Safety of Anacetrapib in Patients with or at High Risk for Coronary Heart Disease. N. Engl. J. Med..

[51] Sahebkar A., Watts G. (2013). New LDL-Cholesterol Lowering Therapies: Pharmacology, Clinical Trials, and Relevance to Acute Coronary Syndromes. Clin. Ther..

[52] Cuchel M., Meagher E.A., Theron H.D.T., Blom D.J., Marais A.D., Hegele R.A., Averna M., Sirtori C.R., Shah P.K., Gaudet D. (2013). Efficacy and safety of a microsomal triglyceride transfer protein inhibitor in patients with homozygous familial hypercholesterolaemia: A single-arm, open-label, phase 3 study. Lancet.

[53] Raal F.J., Giugliano R., Sabatine M.S., Koren M.J., Blom D., Seidah N., Honarpour N., Lira A., Xue A., Chiruvolu P. (2016). PCSK9 inhibition-mediated reduction in Lp(a) with evolocumab: An analysis of 10 clinical trials and the LDL receptor’s role. J. Lipid Res..

[54] Reiner Ž. (2019). Can Lp(a) Lowering Against Background Statin Therapy Really Reduce Cardiovascular Risk?. Curr. Atheroscler. Rep..

[55] O’Donoghue M.L., Fazio S., Giugliano R.P., Stroes E.S., Kanevsky E., Gouni-Berthold I., Im K., Pineda A.L., Wasserman S.M., Češka R. (2019). Lipoprotein(a), PCSK9 Inhibition, and Cardiovascular Risk. Circulation.

[56] Lansberg P.J., Banerjee Y. (2017). A Highly Durable RNAi Therapeutic Inhibitor of PCSK9. N. Engl. J. Med..

[57] Ray K.K., Landmesser U., Leiter L.A., Kallend D., Dufour R., Karakas M., Hall T., Troquay R.P., Turner T., Visseren F. (2017). Inclisiran in Patients at High Cardiovascular Risk with Elevated LDL Cholesterol. N. Engl. J. Med..

[58] Heigl F., Hettich R., Lotz N., Reeg H., Pflederer T., Osterkorn D., Osterkorn K., Klingel R. (2015). Efficacy, safety, and tolerability of long-term lipoprotein apheresis in patients with LDL- or Lp(a) hyperlipoproteinemia: Findings gathered from more than 36,000 treatments at one center in Germany. Atheroscler. Suppl..

[59] Feingold K., Grunfeld C. (2017). Lipoprotein apheresis. Cardiol. Clin..

[60] Virani S.S., Brautbar A., Davis B.C., Nambi V., Hoogeveen R., Sharrett A.R., Coresh J., Mosley T.H., Morrisett J.D., Catellier D.J. (2012). Associations Between Lipoprotein(a) Levels and Cardiovascular Outcomes in Black and White Subjects. Circulation.

[61] Sinzinger H., Steiner S., Derfler K. (2017). Pleiotropic effects of regular lipoprotein-apheresis. Atheroscler. Suppl..

[62] Drouin-Chartier J.-P., Tremblay A.J., Bergeron J., Pelletier M., Laflamme N., Lamarche B., Couture P. (2016). Comparison of two low-density lipoprotein apheresis systems in patients with homozygous familial hypercholesterolemia. J. Clin. Apher..

[63] Morawietz H., Goettsch W., Brux M., Reimann M., Bornstein S.R., Julius U., Ziemssen T. (2013). Lipoprotein apheresis of hy-percholesterolemic patients mediates vasoprotective gene expression in human endothelial cells. Atheroscler. Suppl..

[64] Stulnig T.M., Morozzi C., Reindl-Schwaighofer R., Stefanutti C. (2019). Looking at Lp(a) and Related Cardiovascular Risk: From Scientific Evidence and Clinical Practice. Curr. Atheroscler. Rep..

[65] Stefanutti C., Vivenzio A., Di Giacomo S., Mazzarella B., Ferraro P., Abbolito S. (2010). Treatment of symptomatic hyperLp(a)lipidemia with LDL-apheresis vs. usual care. Transfus. Apher. Sci..

[66] Julius U., Tselmin S., Schatz U., Fischer S., Birkenfeld A.L., Bornstein S.R. (2019). Actual situation of lipoprotein apheresis in patients with elevated lipoprotein(a) levels. Atheroscler. Suppl..

[67] Yu R.Z., Graham M.J., Post N., Riney S., Zanardi T., Hall S., Burkey J., Shemesh C.S., Prakash T.P., Seth P.P. (2016). Disposition and Pharmacology of a GalNAc3-conjugated ASO Targeting Human Lipoprotein(a) in Mice. Mol. Ther. Nucleic Acids.

[68] Wu M.F., Xu K.Z., Guo Y.G., Yu J., Wu Y., Lin L.M. (2019). Lipoprotein(a) and Atherosclerotic Cardiovascular Disease: Current Understanding and Future Perspectives. Cardiovasc. Drugs Ther..

[69] Tsimikas S., Karwatowska-Prokopczuk E., Gouni-Berthold I., Tardif J.-C., Baum S., Steinhagen-Thiessen E., Shapiro M.D., Stroes E.S., Moriarty P.M., Nordestgaard B.G. (2020). Lipoprotein(a) Reduction in Persons with Cardiovascular Disease. N. Engl. J. Med..

[70] Clarke R., Peden J.F., Hopewell J.C., Kyriakou T., Goel A., Heath S.C., Parish S., Barlera S., Franzosi M.G., Rust S. (2009). Genetic Variants Associated with Lp(a) Lipoprotein Level and Coronary Disease. N. Engl. J. Med..

